# The genetic interactions between non-alcoholic fatty liver disease and cardiovascular diseases

**DOI:** 10.3389/fgene.2022.971484

**Published:** 2022-08-10

**Authors:** Nicholas W.S. Chew, Bryan Chong, Cheng Han Ng, Gwyneth Kong, Yip Han Chin, Wang Xiao, Mick Lee, Yock Young Dan, Mark D. Muthiah, Roger Foo

**Affiliations:** ^1^ Department of Cardiology, National University Heart Centre, Singapore, Singapore; ^2^ Yong Loo Lin School of Medicine, National University Singapore, Singapore, Singapore; ^3^ Cardiovascular Research Institute, Yong Loo Lin School of Medicine, National University of Singapore, Singapore, Singapore; ^4^ Cardiovascular Disease Translational Research Programme, National University Health Systems, Singapore, Singapore; ^5^ Genome Institute of Singapore, Agency of Science Technology and Research, Bipolis way, Singapore; ^6^ Division of Gastroenterology and Hepatology, Department of Medicine, National University Hospital, Singapore, Singapore; ^7^ National University Centre for Organ Transplantation, National University Health System, Singapore, Singapore

**Keywords:** genetics, cardiovascular diseases, non-alcoholic fatty liver disease, coronary artery disease, cardiomyopathy, atrial fibrillation

## Abstract

The ongoing debate on whether non-alcoholic fatty liver disease (NAFLD) is an active contributor or an innocent bystander in the development of cardiovascular disease (CVD) has sparked interests in understanding the common mediators between the two biologically distinct entities. This comprehensive review identifies and curates genetic studies of NAFLD overlapping with CVD, and describes the colinear as well as opposing correlations between genetic associations for the two diseases. Here, CVD described in relation to NAFLD are coronary artery disease, cardiomyopathy and atrial fibrillation. Unique findings of this review included certain NAFLD susceptibility genes that possessed cardioprotective properties. Moreover, the complex interactions of genetic and environmental risk factors shed light on the disparity in genetic influence on NAFLD and its incident CVD. This serves to unravel NAFLD-mediated pathways in order to reduce CVD events, and helps identify targeted treatment strategies, develop polygenic risk scores to improve risk prediction and personalise disease prevention.

## Introduction

Non-alcoholic fatty liver disease (NAFLD) is characterised by the condition of excess fat in the liver in the absence of excessive alcohol consumption (Ludwig et al., 1980). It is frequently associated with metabolic syndrome, affecting at least a quarter of the population worldwide (Younossi et al., 2016). NAFLD is a disease spectrum consisting of non-alcoholic fatty liver (NAFL–i.e., hepatic fat accumulation), non-alcoholic steatohepatitis (NASH), fibrosis and cirrhosis (Kleiner et al., 2005). The main risk factors for NAFLD include hyperlipidaemia, diabetes mellitus, hypertension and obesity (Rinella, 2015), which are also key risk factors for cardiovascular disease (CVD) (Liu and Lu, 2014). The causal relationship between NAFLD and CVD is therefore of particular interest, although the pathogenesis of both is widely agreed to involve the complex interplay between genetic and environmental factors (Liu and Lu, 2014). Liver fat accumulation results from an imbalance between the influx of fat which includes fatty acids from adipose tissue and *de novo* lipogenesis from glucose, and fat efflux which involves oxidation and synthesis of very-low-density lipoprotein (VLDL) (Brouwers et al., 2020). Even though liver fat accumulation is the prerequisite for the progression of NASH, not all NAFL individuals progress through the stages. Lipotoxicity is a main driver of NASH progression, determined by the quantity and type of accumulated lipids and the defensive capabilities of the liver against lipotoxicity (Donnelly et al., 2005; Machado and Diehl, 2016). Apart from the natural history of NASH progression, our group was also able to depict significant improvement in liver histology in the absence of pharmacological interventions, suggesting that NASH is a disease that can regress spontaneously over time with even non-pharmacological measures (Ng et al., 2022).

NAFLD appears to be directly associated with CVD, independent of traditional cardiovascular confounders such as age, sex, state of hyperglycaemia and insulin resistance (Stefan et al., 2016). It is strongly associated with an increased risk of incident CVD (Targher et al., 2010; Anstee et al., 2013; Armstrong et al., 2014; Ballestri et al., 2014; Byrne and Targher, 2015; Lonardo et al., 2015), as well as a 64% higher risk of developing non-fatal and/or fatal CVD, compared to non-NAFLD individuals (Ong et al., 2008; Targher et al., 2016). Unsurprisingly, even though patients with NAFLD are at risk of end-stage liver disease, the majority of NAFLD patients die from CVD (Targher et al., 2010; Chalasani et al., 2012; Anstee et al., 2013).

The ongoing debate of whether NAFLD is an active contributor or an innocent bystander in the development of CVD therefore encompasses the appreciation for the common mediators between the two anatomically distinct entities. Exemplifying potential shared mediators between NAFLD and CVD is the pathogenesis of atherosclerotic lesions, which is the combination of endothelial dysfunction, lipoprotein accumulation in the vessel wall, inflammatory cell infiltrates, accumulation of foam cells and proliferation of the smooth muscle cells resulting in vulnerable plaques that are prone to rupture (Brouwers et al., 2020). NAFLD could contribute theoretically to all these pathophysiological stages, through the development of dyslipidaemia (characterised by elevated plasma triacylglycerols, low levels of high-density lipoprotein (HDL) cholesterol, high levels of small-dense low-density lipoprotein (LDL) particles (Adiels et al., 2006; Adiels et al., 2008; Defilippis et al., 2013; Do et al., 2013; Holmes et al., 2017; Burgess et al., 2018), low-grade inflammation (involving IL-1β in the pathogenesis of NASH and CVD) (Ridker et al., 2017; Mirea et al., 2018), and thrombogenicity (Alessi et al., 2003; Song et al., 2017) (with high levels of plasminogen activator inhibitor type-1 [PAI-1] in NASH, that is also an important component in the fibrinolytic system). Some NAFLD susceptibility genes are associated with CVD, representing possible components mediating between both entities. A systematic analysis of the human genetics for both conditions may therefore offer important therapeutic strategies to treat NAFLD and offset the greater CVD risk. Simple steatosis indeed is not as ‘simple’ or benign as its name suggests, with fat accumulation driving both the overproduction of lipoproteins and atherosclerosis. Whilst NAFLD and CVD share similar susceptibility genes, certain genes have displayed opposing correlations between polymorphism and the 2 different diseases. For example, as both the phospholipase domain-containing protein 3 (*PNPLA3*) and transmembrane 6 superfamily 2 (*TM6SF2*) regulate VLDL particle production, the genetic polymorphism can result in increased intrahepatic triglyceride (TG) with increased risk of NAFLD, but reduced circulatory cholesterol and LDL which lends protection against coronary artery disease (CAD) (Liu et al., 2017; Simons et al., 2017). Delineating these unique pathways will be important in unravelling NAFLD-mediated pathways in order to reduce CVD events, and facilitate future studies in identifying targeted treatment strategies (Brouwers et al., 2020).

This review aims to give insight into the genetic interactions between CVD and NAFLD, and elaborate on the experimental evidence that supports a causal relationship between the two entities. We discuss the clinical implications of the findings, which may guide future therapeutic trials targeting common genetic pathways in order to simultaneously lower the risk of NAFLD and CVD. Here, CVD described in relation to NAFLD are CAD, cardiomyopathy and atrial fibrillation (AF).

## NAFLD and coronary artery disease

Genome-wide genotyping arrays have from the start contributed to the large spectrum of genetic variants with subsequent imputation of millions of further variants leading to a wide breadth of understanding of the genetic architecture of CAD (Khera and Kathiresan, 2017; Erdmann et al., 2018). Ultra-large scale genome-wide association studies (GWASs) and meta-analyses report loci in relation to atherosclerosis, demonstrating the progressive insights for population-specific and trans-ancestry risk factors in CAD (Lu et al., 2012; Takeuchi et al., 2012; Koyama et al., 2020). This is proving utility for polygenic risk scores in CAD or myocardial infarction (MI), depending on the accuracy of predicting the effect size of risk alleles that varies with individual genetics (Gola et al., 2020). More than 200 loci have been associated with CAD and MI since 2007, with comprehensive identification of their risk alleles, and allele frequencies (Erdmann et al., 2018; Kessler and Schunkert, 2022). Initially, the impact of genetic hits lay with traditional cardiovascular risk factors: hypertension and hyperlipidaemia. Currently, approximately half of the loci explains the impact on pathophysiological pathways (such as nitric oxide signalling), and others are identified as new “drivers” of atherosclerosis and MI (Wobst et al., 2018; Dang et al., 2020). Several NAFLD susceptibility genes have a differential effect on plasma lipids and concurrent CAD risks, suggesting that plasma lipids are indeed essential mediators between NAFLD and CVD. This may guide the design of anti-NAFLD drugs targeting lipid metabolism and CAD risks (Brouwers et al., 2020). A summary of genomic variants overlapping between NAFLD and CVD is shown in Table 1.

**TABLE 1:** Summary of genomic variants identified in both NAFLD and CVD.

Gene	Protein function	Association with NAFLD	Association with cardiovascular diseases
Apolipoprotein C-3 (*APOC3*)	Lowers lipoprotein lipase activity, inhibits triglyceride hydrolysis into VLDL particles and chylomicrons in the plasma, thus increasing plasma triglycerides → increased CAD risk	• *C482T* and *T455C* variants experience a 30% higher fasting plasma APOC3 and 60% higher fasting plasma triglycerides in the presence of NAFLD.^41^	• Rare mutations that disrupt the *APOC3* function were associated with 39% lower levels of plasma triglycerides compared to noncarriers, and a 40% reduction in CAD risks.^40^
		• When compared to subjects with the wild-type allele, 38% of the *C482T* and *T455C* carriers had NAFLD.^41^	• Heterozygous carriers of the null mutation of the gene encoding *APOC3* expressed half the amount of *APOC3* than noncarriers. Leading to lower fasting and postprandial serum triglycerides, lower levels of LDL-cholesterol and higher levels of HDL-cholesterol. Subclinical atherosclerosis was less common in carriers than noncarriers. Lifelong deficiency of APOC3 has a cardioprotective impact on carriers.^39^
Apolipoprotein E (*APOE*)	Plasma lipid transport protein that facilitates clearance of triglyceride-rich lipoproteins from bloodstream into the liver.	• Prevalence of the APOE ε3 allele^87^ is significantly increased in biopsy-proven NASH patients	• Hypolipidemic effect of APOE ε2 leads to lower LDL and higher HDL concentrations in childhood.^82,83^
		• Occurrence of APOE ε2 allele is protective against NAFLD.^89^	• APOE ε4 isoform shown to be an indicator for CVD risk, with associations of increased carotid intima-media thickness, LDL, Lp(a) and apoB levels.^84,85^ Dysbetalipoproteinemia may arise.
Insulin receptor substrate-1 (*IRS-1*)	Regulates insulin action downstream, and maintains vascular smooth muscle cell differentiation.	• *Gly172Arg* polymorphism alters the activity of insulin receptors and increases the severity of NAFLD in Caucasians.^117^	• Genetic variation near *IRS-1* results in decreased IRS-1 expression. This is associated with impaired metabolic profile - increased visceral to subcutaneous fat ratio, insulin resistance, hyperlipidaemia, decreased adiponectin levels → enhancing the risk of diabetes and CAD.^109,110^
Phosphatidylethanolamine N-methyltransferase (*PEMT*)	Catalyses the conversion of Phosphatidyethanolamine to phosphatidylcholine. Phosphatidylcholine contributes to VLDL formation for hepatic triglyceride secretion.	• The V175Met loss-of-function mutation confers susceptibility to NASH,^106^ in both the Chinese^107^ and Japanese population.^100^	• V175Met is a loss of function mutation, associated with diminished PEMT activity, that increases propensity to lipid accumulation.^96^
		• Val175Met variant allele of the PEMT gene is significantly more prevalent in NASH patients.	• Carriers of the PEMT risk allele showed decreased levels of multiple glycerophospholipids such as the cardioprotective lipid species LPC 16:0 and/or LPC 20:4.^95^
		• In the NASH group, carriers of Val175Met had significantly lower body mass index and more non-obese patients compared to homozygotes of wild type PEMT.^100^	
*TRIB1*	Regulatory mechanisms on circulatory lipids and immune cells. It reduces TG, maintains steady-state phagocytosis and M2 macrophage differentiation, and inhibits chemotaxis of inflammatory factors and vessel wall damage	• TRIB1 knockout reduces expression of MTTP and APOB (the main apolipoprotein component of VLDL and LDL.	• Risk allele at the TRIB1 locus was associated with higher triglyceride and lower HDL-cholesterol.^51,62,63^
		• TRIB1 knockout increases expression of liver C/EBPα protein, thus increases production and accumulation of hepatic fat	
Tumor necrosis factor-a (*TNF-a*)	Acts as a pro-inflammatory cytokine. It has a broad role in inflammation, autoimmunity, tumour apoptosis and metabolic dysregulation	• Higher prevalence of *TNF-α -238* promotor polymorphism in NAFLD/NASH patients, in Italian,^176^ Mexican^177^ and Chinese^107,178^ cohorts.	• *TNF-a*/-308 A allele overexpressed in patients with end-stage non-ischemic DCM.^170^
		• *TNF-α* -238 polymorphism associated with inverse effect on regulation of glucose and lipid homeostasis, resulting in increased risk of impaired insulin sensitivity, but lower LDL-cholesterol and BMI	• *TNF-a/*-308 A allele was more prevalent in Japanese patients with idiopathic DCM.^167^
			• Frequencies of *TNF-a*/-238 (G/A) alleles in the Turkish cohort differed from those in the France and Northern Ireland cohorts.^170^
Interleukin 6 (*IL 6*)	Acts as a pro-inflammatory cytokine	• Prevalence of IL6-174C variant was higher in NASH than NAFLD patients in a Caucasian population and was associated with increased insulin resistance.^189^	•Interleukin-6 (IL6) is significantly correlated with increased left atrial size,^184^ an important predictor for new onset AF.
			• IL6 stimulates matrix metalloproteinase-2, a molecule implicated in the atrial remodelling process in AF.^185,186^
			• Polymorphisms in the promotor region of the *IL6* gene correlates with postoperative AF.^187,188^
			• CC genotype of the *IL6*–*174 G/C IL6* polymorphism showed significant elevation of IL6 levels and has at least 2 fold increase in AF incidence compared to GC and GG genotypes.
Patatin-like phospholipase domain-containing protein 3 (*PNPLA3*)	Lipid droplet remodelling and VLDL production. The PNPLA3 protein reduces hydrolyse activity, leading to impaired intrahepatic TG breakdown and hampering VLDL particle production and secretion.	• *PNPLA3* rs738409 G allele predisposes to NAFLD.^141,142^	• *PNPLA3* rs738409 G allele conferred a modest protection from CAD.^141,142^
		• The I148M variant of *PNPLA3* associated with NAFLD severity. It increases risk of developing NASH, advanced fibrosis and cirrhosis.^146^ This has been observed in the Japanese, Italians, Malaysians and Americans^147-151^ populations.	• Variants in *PNPLA3* associated with lower plasma triacylglycerols and LDL cholesterol^142^ → reducing risk of CAD
			• *PNPLA3* rs738409 G allele encodes PNPLA3 protein (I148M) which reduces hydrolyse activity, leading to impaired intrahepatic TG breakdown, hampers VLDL particle production and secretion.^144^ Therefore, *PNPLA3* minor allele correlated with reduction in plasma lipids^145^ → reducing risk of CAD
Transmembrane 6 superfamily 2 (*TM6SF2*)	Regulates liver fat metabolism, TG secretion and hepatic lipid droplet content.^154^ It is involved in VLDL production.	• The rs5854926 variant reduces *TM6SF2* expression in the liver, resulting in higher serum alanine aminotransferase levels and TG content, and reduced total cholesterol and LDL.^155-158^	• *TM6SF2* rs58542926 T allele has cardioprotective effects.^141,153^
		• *TM6SF2* rs5854926 variant has been shown to be an independent risk factor for liver steatosis.^155,156,160^	• Variants of the gene associated with reduced plasma LDL-cholesterol and triacylglycerols^142^ → reduces risk of CAD
		• *TM6SF2* rs5854926 variant significantly associated with NAFLD in Asian and Caucasian populations.^161^	
Glucokinase regulatory protein (*GCKR*)	•Regulates glucose storage and disposal.	• rs1260326-T variant decreases GCKR ability to inhibit glucokinase → increased hepatic glucose uptake, decreased fatty acid oxidation and enhanced lipogenesis → the progression of steatosis or levels of circulating lipids.	• Variants (rs1260326, rs780094, rs780093) associated with CAD,^119^ higher serum triacylglycerols, lower serum HDL cholesterol and the presence of small dense LDL particles.^118^
	•Encodes liver-specific glucokinase regulatory protein (GKRP), which has role in *de novo* lipogenesis.	• rs1260326-T variant increases NAFLD risks and the concentrations of apolipoprotein B which contains lipoprotein particles and TG.^127,128^	
		• rs789904-T variant associated with hepatic steatosis.^131^	

VLDL, Very low-density lipoprotein; LDL, Low density lipoprotein; HDL, High density lipoprotein; TG, Triglycerides; AF, Atrial fibrillation; CAD, Coronary artery disease; CVD, Cardiovascular disease; NAFLD, Non-alcoholic fatty liver disease; NASH, non-alcoholic steatohepatitis.

### Apolipoprotein C-3

Lipoprotein lipase (LPL) is an essential enzyme for metabolising TG-rich lipoproteins. Populations harbouring common, noncoding, and rare loss of function variants at this genetic locus, have been associated with elevated CAD risk. In addition to the *LPL* gene, crucial endogenous regulators of LPL activity are associated with CAD, including *APOC3* (Pollin et al., 2008; Willer et al., 2008; Crosby et al., 2014). *APOC3*, expressed in the liver, is a key regulator of plasma TG levels, shown to adversely affect cardiovascular event risks. *APOC3* lowers LPL activity, and inhibits TG hydrolysis into VLDL particles and chylomicrons in the plasma, increasing plasma TG and the resultant increased risk of CAD. Rare mutations disrupting APOC3 function are associated with 39% lower plasma TG compared to noncarriers, as well as a 40% reduction in CAD risks (Crosby et al., 2014). 5% of heterozygous carriers of the null gene *APOC3* mutation express half the amount of *APOC3* in noncarriers. Mutation carriers have lower fasting, postprandial serum TG, as well as lower levels of LDL-cholesterol and higher HDL-cholesterol. As a result, subclinical atherosclerosis, determined by coronary artery calcification, were less common in carriers than noncarriers. This proposes that lifelong deficiency of *APOC3* provides a cardioprotective impact on carriers (Pollin et al., 2008).

Polymorphisms in *APOC3* are also associated with NAFLD and insulin resistance. In a cohort of Asian Indian men (Cooper, 1985; Weintraub et al., 1987; Petersen et al., 2010), carriers of *APOC3* variant alleles (*C-482T, T-455C*, or both) demonstrated a 30% increase in fasting plasma apolipoprotein C3 concentration, compared to wild-type homozygotes. *APOC3* variant carriers had 60% increased fasting plasma TG concentration. Increased plasma APOC3 concentration inhibits LPL and TG clearance, predisposing to increased fasting and postprandial hypertriglyceridemia as a result of increased chylomicron-remnant particles. Elevated concentrations of circulating chylomicron-remnant particles are taken up by the liver through a receptor-mediated process, leading to NAFLD and hepatic insulin resistance. Moreover, increased hepatocellular diacylglycerol concentrations in NAFLD result in protein kinase C epsilon isoform activation that reduces insulin signalling and hepatic insulin resistance (Samuel et al., 2004; Zhang et al., 2007). 38% of subjects with *APOC3* variant alleles had NAFLD, compared to 0% among wild-type homozygotes, and those with NAFLD had marked insulin resistance. This observation was also confirmed in a second cohort of non-Asian Indian men (Petersen et al., 2010). Interestingly, other cohorts of European ancestry (Kozlitina et al., 2011; Sentinelli et al., 2011; Hyysalo et al., 2012) did not identify any significant association between *APOC3* polymorphisms, NAFLD and insulin resistance.

In the current therapeutic pipeline, selective antisense *APOC3* inhibitor, *Volanesorsen*, produces a dose-dependent 31–71% decrease in TG, used for targeting TG reduction in familial chylomicronemia syndrome (Pollin et al., 2008; Willer et al., 2008; Crosby et al., 2014; Gaudet et al., 2015). A randomised, placebo-controlled phase 2 trial demonstrated dose-dependent and prolonged decrease in levels of plasma APOC3 when the drug was administered as a monotherapy or as an add-on to fibrates. Studies are needed to examine whether TG reduction via antisense *APOC3* inhibition translates also to improved liver-related outcomes given the close links between hepatic lipoprotein metabolism and NAFLD (Heeren and Scheja, 2021).

### TRIB1

The *TRIB1* gene encodes for the Tribbles homolog 1 protein, which is part of a family of phosphoproteins that play a role in cell function regulation. The single nucleotide polymorphisms (SNPs) in or near *TRIB1* (*such as* rs17321515) have demonstrated significant associations with increased circulatory triglycerides and CAD. Another promising target with associations for cardiovascular traits is the recently identified *TRIB1* locus (Teslovich et al., 2010; IBC 50K CAD Consortium, 2011; Deloukas et al., 2013). Regulatory mechanisms associated with *TRIB1* on circulatory lipids and immune cells support the notion that *TRIB1* is associated with the development of CVD. Atherosclerosis develops from vascular wall damage, endothelial cell dysfunction or death, inflammatory cytokine and chemokine production, oxidised LDL particles, formation of foam cells, and subsequently plaque formation (Jaipersad et al., 2014; Grootaert et al., 2018). In atherosclerosis pathophysiology, macrophages undergo M2 to M1 transdifferentiation (Deloukas et al., 2013). *TRIB1* has been associated with M2 macrophage transition, offering an important role in homeostatic maintenance and repair of damaged tissue (Khallou-Laschet et al., 2010; Baitsch et al., 2011; Satoh et al., 2013). Hence *TRIB1* knockout impairs M2 macrophage mediated repair, leading to metabolic disease and CVD. Furthermore, mitogen-activated protein kinase (MAPK) mediates inflammatory factor-mediated migration and vascular smooth muscle cell proliferation. TRIB1 negatively regulates MAPK activity by binding to MAPK kinase, thus reducing the chemotaxis of inflammatory factors and inhibiting vascular smooth muscle cell migration and proliferation (Manichaikul et al., 2012; Wang et al., 2015; Pan, 2017; Reustle and Torzewski, 2018).

Data from GWA meta-analyses (Zeggini et al., 2008; Newton-Cheh et al., 2009; Teslovich et al., 2010) demonstrated that the risk allele at the *TRIB1* locus is associated with higher TG and lower HDL-cholesterol. *TRIB1* affects lipid metabolism with an inverse correlation between hepatocellular *TRIB1* expression and lipogenic gene expression. Thus *TRIB1* expression reduces the risk of CVD through the reduction of TG, maintenance of steady-state phagocytosis, percentages of M2 macrophages, and inhibiting the chemotaxis of inflammatory factors and vessel wall damage (Zhang et al., 2021). Animal studies show that *TRIB1* overexpression in wild-type mice lowered circulating TG levels (Burkhardt et al., 2010); conversely, targeted *TRIB1* deletion in mice led to higher circulating TG. The risk of CAD is thus potentially downstream of *TRIB1*, which is likely to be mediated by the regulation of *TRIB1* expression resulting in adverse lipid profiles (The, 2011). A study in the Chinese Han population (Liu et al., 2019a) demonstrated that *TRIB1* rs17321515 AA + GA genotypes were significantly associated with CAD in the general population as well as in the NAFLD population, despite adjusting for important confounders. Moreover, *TRIB1* rs17321515-A carriers displayed worse lipid profiles than non-carriers. *TRIB1* rs17321515 GA + AA genotypes and *TRIB1* rs2954029 TA + AA genotypes also independently increased NAFLD risk in the Chinese Han population (Liu et al., 2019b).

*TRIB1* overexpression in mouse liver decreases plasma cholesterol, TG and VLDL secretion (Bauer et al., 2015; Soubeyrand et al., 2016). Together with Sin3A associated protein 18 (SAP18) and mSin3A, TRIB1 activates the transcription of microsomal triglyceride transporter protein (Makishima et al., 2015) (*MTTP*). Hence *TRIB1* knockout in human hepatic cells reduces the expression of *MTTP* and *APOB* (Burkhardt et al., 2010; Makishima et al., 2015; Nagiec et al., 2015) (which is the main apolipoprotein component of VLDL and LDL). Conversely, *TRIB1* has a functional role in lipogenesis, where *TRIB1* overexpression downregulates the carbohydrate response element binding protein (ChREBP), which is a glucose-sensitive molecule important for hepatic lipogenesis (Iwamoto et al., 2015; Softic et al., 2017). *TRIB1* knockout increases the expression of liver C/EBPα protein, thus increasing the production and accumulation of hepatic fat, resulting in liver cell damage, liver steatosis and NAFLD (Bauer et al., 2015; Kahali et al., 2015). Further studies on this treatment approach being transferred to human treatment can be the next important step (Kessler and Schunkert, 2021).

### Apolipoprotein E

Apolipoprotein E (ApoE) is a constituent of lipoproteins with large variations due to cysteine-arginine exchanges. *APOE* gene variants give rise to apoE isoforms with six permutations of the apoE or ε variants–E2/2, E2/3, E2/4, E3/3, E3/4, E4/4 (Mahley, 2001). Common apoE variants account for approximately 4% of the variance in plasma cholesterol. Another variant of apoE (rs35136575) also affects LDL concentrations (Boerwinkle and Utermann, 1988).

ApoE facilitates the clearance of TG-rich (apoB-containing) lipoprotein remnants from the bloodstream into the liver. It affects vascular function through mechanisms such as inflammatory responses, platelet aggregation and inflammatory effects involving M2 macrophage phenotype transformation and proliferation of lymphocytes and T helper cells (Riddell et al., 1997; Baitsch et al., 2011; Zhang et al., 2011). As such, the apoE isoforms influence CVD risk from birth–for example, the hypolipidemic effect of apoE2 can be seen from childhood with lower LDL and higher HDL concentrations (Kallio et al., 1997; Isasi et al., 2000). The apoE4 isoform is associated with increased carotid intima-media thickness, LDL, Lp(a) and apoB levels (Granér et al., 2008; Luo et al., 2017). The cardiovascular effect of common variants is observed from its influence on the lipid profile with the potential of more severe and pathological sequelae. Dysbetalipoproteinemia arises in middle-aged males and post-menopausal females, and remnant lipoproteins accumulate due to impaired clearance or overproduction of lipoproteins. TG accumulation in dysbetalipoporteinemia increases overall atherogenicity. Dominantly inherited mutations offer the diagnosis of familial hypercholesterolemia (Marais, 2019).

In NASH patients, there was an increased prevalence of the *APOE* e3 allele compared to healthy controls: *APOE* polymorphism was significantly associated with NASH, particularly the *APOE*3/3 genotype (Sazci et al., 2008). In animal studies, loss of ApoE reduced susceptibility to obesity and NAFLD (Naik et al., 2013). The *APOE* e3 allele (Sazci et al., 2008) is also significantly more prevalent in biopsy-proven NASH patients compared to controls, whereas the APOE e2 allele appears protective against NAFLD (Demirag et al., 2007). Currently, the *APOE*3/3 genotype is recognised to play a role in the aetiopathogenesis of NASH (Sazci et al., 2008). Larger studies are needed to examine this association across different ethnicities.

Arising from the above, some have proposed genetic testing of common variants for clinical screening, with apoE2 protective against and apoE4 at increased risk of vascular disease (Jacobsen et al., 2010; Zhao et al., 2017). ApoE may provide novel therapeutic targets for the treatment of atherosclerosis. The apoE mimetic peptide EpK enhances cholesterol efflux from cells and has beneficial effects in animal model of atherosclerosis (Zhao et al., 2011). Another hepatic-expressed peptide, hEp, lowers lipoprotein levels and also protects against atherosclerosis (Xu et al., 2016). An individual’s apoE status may indeed provide valuable insight into cardiovascular health, on the backdrop of hyperlipidaemia and CVD. Furthermore, the *APOE* genotype may also affect response to statin therapy, with the largest HDL increase in apoE2 carriers. Fibrates produces the largest decrease in TG in apoE2 carriers, and the slowest effect in apoE4 carriers (Buckley et al., 2004).

### Phosphatidylethanolamine N-methyltransferase

Many CAD susceptibility genes are associated with concentrations of circulating lipid species. One locus, phosphatidylethanolamine N-methyltransferase (*PEMT*), encodes for the enzyme for lipid biosynthesis (Fernandez et al., 2013). PEMT catalyses all three methylation steps in the conversion of phosphatidyethanolamine to phosphatidylcholine, using S-adenosylmethionine as a methyl group donor. *PEMT is largely expressed in the liver and accounts for 30% of liver* phosphatidylcholine production (Vance, 2014). A functional SNP can lead to the loss of function with amino acid replacement Val175Met in *PEMT* which alters the catalytic function for the conversion of phosphatidyethanolamine to phosphatidylcholine. Phosphatidylcholine is important for VLDL formation for hepatic TG secretion. The V175M results in loss of function, associated with diminished PEMT activity, with increased propensity to lipid accumulation (Noga et al., 2002). Carriers of the *PEMT* risk allele indeed show decreased levels of multiple glycerophospholipids, such as the cardioprotective lipid species LPC 16:0 and/or LPC 20:4 (Fernandez et al., 2013). Here, integrative analysis of genomics with lipidomics advanced the insights for underlying mechanisms and pathogenesis of CVD.

In the liver, the PEMT catalyses three methylation steps in the conversion of phosphatidyethanolamine to phosphatidylcholine (Browning and Horton, 2004). The fast onset of liver cell damage in a methionine and choline deficient diet, and liver-specific expression of PEMT may be attributed to the high demand for choline and phosphatidylcholine (Browning and Horton, 2004) that is important for the maintenance of normal liver function (Vance and Ridgway, 1988; Vance et al., 1997). The PEMT and CDP-choline pathways are two important regulatory pathways in maintaining phosphatidylcholine homeostasis in hepatocytes. Phosphatidylcholine homeostasis is necessary as it is the primary phospholipid of all classes in humans, necessary for VLDL secretion (Vance and Vance, 1985; Yao and Vance, 1988, 1989; Vermeulen et al., 1997; Dong et al., 2007). The extent and rate of TG accumulation in hepatocytes is determined by hepatocyte efficacy for excreting VLDL. In addition, the single nucleotide polymorphism G433T in microsomal TG transfer protein (*MTTP*) gene also influences the degree and rate of fat deposition in hepatocytes (Namikawa et al., 2004). TG, together with cholesterol and phospholipids, assemble together to form apolipoprotein B in hepatocytes and is secreted as VLDL. As such, phosphatidylcholine deficiency and MTTP activity impairment (Yao and Vance, 1988, 1989; Noga et al., 2002) adversely affects VLDL secretion from hepatocytes, causing accumulation in hepatocytes, which is at the core of NASH pathophysiology. Furthermore, aberrant sterol regulatory element-binding proteins (SREBPs) activity can lead to excess stored fat and obesity, through the activation of genes involved in lipid synthesis, trafficking and homeostasis. A study suggested that the maturation of nuclear, transcriptionally active SREBP-1 is influenced by phosphatidylcholine (Walker et al., 2011). Therefore, genetic conditions (such as PEMT Val175Met) that limit phosphatidylcholine production can in turn activate SREBP-1, and as a consequence increasing the size and amount of lipid droplets, and exacerbating the risk of developing metabolic diseases (Walker et al., 2011). A separate study revealed that in PEMT knockout mice, the ratio of phosphatidylcholine/phosphatidylethanolamine is decreased, leading to loss of membrane integrity, which has important clinical implications in the progression of NAFLD (Li et al., 2006). Overall, the V175M loss-of-function mutation in the human *PEMT* gene confers susceptibility to NASH (Song et al., 2005), and this has been shown in both the Chinese (Zhou et al., 2010) and Japanese population (Dong et al., 2007). In the NASH group, Val175Met carriers have significantly lower body mass index (BMI) with more non-obese patients, compared to homozygotes of wild type *PEMT* (Dong et al., 2007). The *PEMT* Val175Met variant may be a potential molecular target for novel NASH therapy.

### Insulin receptor substrate-1

The insulin receptor substrate-1 (IRS-1) gene encodes a protein which is phosphorylated by insulin receptor tyrosine kinase and plays an important role in the insulin-stimulated signal transduction pathway. Both hyperglycaemia and insulin resistance can downregulate IRS-1, which is a pivotal intermediary in insulin/IGF-1 signalling (Xi et al., 2019). IRS-1 is essential for maintaining vascular smooth muscle cell differentiation. Hyperglycaemia or insulin resistance-induced *IRS-1* downregulation decreased p53/Kruppel like factor 4 (KLF4) association and increased dedifferentiation and proliferation of vascular smooth muscle cells. Therefore, enhancing *IRS-1* dependent p53 stabilisation may retard atherosclerosis progression, particularly in individuals with hyperglycaemic or insulin resistance states (Xi et al., 2019). Conversely, genetic variations near *IRS-1* (such as the major allele of rs2972146 (Teslovich et al., 2010), rs2943641 (Rung et al., 2009), rs2943634 (Samani et al., 2007)), resulting in decreased *IRS-1* expression, are associated with impaired metabolic profile, such as an increased visceral to subcutaneous fat ratio, insulin resistance, hyperlipidaemia, decreased adiponectin levels, thus enhancing the risk of diabetes and CAD (Samani et al., 2007; Rung et al., 2009). A GWA meta-analysis explained that there is reduced *IRS-1* expression, associated with the genetic variations near *IRS-1*, in major insulin target tissues such as adipose tissue and muscle. *IRS-1* genetic variations have a deleterious effect on insulin resistance, reduced ability to store subcutaneous fat, and disrupted insulin signalling in liver and muscle, resulting in ectopic deposition of lipids (Stumvoll and Jacob, 1999; Arner, 2002).

Insulin resistance, common co-existing with NAFLD, is contributed by inflammatory factors binding to IRS for ubiquitin-mediated degradation via the activation of Suppressors of Cytokine Signalling 3 (SOCS3). This leads to insulin desensitisation. Studies have shown that the frequency of the Arg allele of the Gly972Arg polymorphism of *IRS-1* gene was significantly increased in NAFLD. Gly972Arg carriers are at significantly higher risk of NAFLD (Bhatt and Guleria, 2021). The association between *IRS-1* gene polymorphism and incident type 2 diabetes has been demonstrated in both the Asian and Caucasian populations (Dongiovanni et al., 2010; Li et al., 2016). The *IRS-1* (Gly972Arg) polymorphism also affects insulin receptor activity, predisposing to hepatocyte injury and decreased hepatic insulin signalling in NAFLD individuals (Dongiovanni et al., 2010). In terms of treatment, diabetic NAFLD patients are treated with PPARγ-agonists thiazolidinedione, which acts as an insulin sensitizer that reduces lipid release and increases lipid uptake, storage and reduces hepatic gluconeogenesis (Vanni et al., 2010). However, polymorphisms in *IRS-1* can affect insulin receptor activity, and can significantly predispose biopsy-proven NAFLD individuals to worse disease severity (Dongiovanni et al., 2010).

### Glucokinase regulatory protein

*GCKR* is a NAFLD susceptibility gene that encodes liver-specific glucokinase regulatory protein (GKRP), which plays an important role in *de novo* lipogenesis and development of NAFLD (Donnelly et al., 2005; Brouwers et al., 2015). Several studies have demonstrated that gene variants (rs1260326, rs780094, rs780093) are associated with CAD (Simons et al., 2018), higher serum triacylglycerols, lower serum HDL cholesterol and the presence of small dense LDL particles (Sookoian and Pirola, 2011; Brouwers et al., 2015; Tabas et al., 2015; Lauridsen et al., 2018). This lipid profile is an example of vertical pleiotropy or mediation, in which the genetic effect on lipids is via the liver given that this lipid phenotype is reported to be the consequence of NAFLD (Defilippis et al., 2013; Brouwers et al., 2015). Furthermore, the metabolic effect of these common variants of *GCKR* can be different in patients with type 2 diabetes, as glucokinase has been shown to be more active when plasma glucose levels are within the diabetic range. This may allude to the difference in effect size of the *GCKR* allele variant on plasma TG being more pronounced in diabetic patients compared to non-diabetic individuals (Agius, 2008; Liu et al., 2012). In fact, Nynke Simons and colleagues (Simons et al., 2016) have reported that rs1260326 interacts with indices of glucose metabolism (such as fasting plasma glucose, Hba1c, glucose tolerance states) which are prominent components of diabetic dyslipidaemia (Taskinen, 2003). Those with moderately controlled diabetes (Hba1c 8.0%) carrying 2 T alleles tended to have higher plasma TG levels compared to homozygous carriers of the C allele, whilst no differences were noted in healthy individuals (Simons et al., 2016).

The molecular pathways that give rise to fatty liver involves excessive hepatic glucose levels and increased lipogenesis (Beer et al., 2009). *GCKR* rs1260326-T associates with decreased GCKR activity to inhibit glucokinase, leading to increased hepatic glucose uptake, decreased fatty acid oxidation and enhanced lipogenesis. Hepatic fatty acids can either be converted to TG and stored in hepatic lipid droplets, or secreted in VLDL particles (Beer et al., 2009; Hodson and Frayn, 2011). Therefore, decreased GCKR activity contributes to the progression of steatosis or levels of circulating lipids. In line with this observation, *GCKR* rs1260326-T increases NAFLD risk and increases the concentrations of apolipoprotein B which contains lipoprotein particles and TG. Moreover, *GCKR* rs1260326-T is associated with increased glycolysis-related metabolites, circulating fatty acids and elevated fatty acid saturation, in combination with the increased overall glycolytic and lipogenic activities (Beer et al., 2009; Rees et al., 2012; Santoro et al., 2015). Moreover, in a large GWAS, *GCKR* rs789904-T was associated with hepatic steatosis diagnosed by computed tomography and biopsy-proven NASH involving lobular inflammation and fibrosis (Speliotes et al., 2011). Moreover, *GCKR* rs1260326-T was recognised as an important factor for inter-individual variation in liver fat (Santoro et al., 2012; Dongiovanni et al., 2015b; Goffredo et al., 2016).

There is always a theoretical concern regarding the development of drugs, with the potential adverse effect of worsening NAFLD. The common *GCKR* variant has a more pronounced effect on hepatic fat accumulation and plasma triacylglycerols in individuals with obesity (Simons et al., 2016; Stender et al., 2017) and hyperglycaemia. It is plausible that individuals with obesity or poorly controlled diabetes may be more prone to adverse effects of liver-specific glucokinase activators, which increase hepatic glucose intake, leading to increased accumulation of hepatic adiposity through enhanced *de novo* lipogenesis (Lloyd et al., 2013; Hodson and Brouwers, 2018; Zhu et al., 2018). Studies are needed to provide greater clarity on these potential adverse effects due to the intertwined effects of NAFLD, plasma lipid levels and CAD risks.

### Patatin-like phospholipase domain-containing protein 3

Another NAFLD susceptibility gene *PNPLA3* has been shown to associate with all stages of NAFLD (Romeo et al., 2008; Sookoian and Pirola, 2011). The common variant *PNPLA3* rs738409 was studied in a Mendelian randomisation (MR) study to evaluate the causal relationship between NAFLD and CAD (Lauridsen et al., 2018). Several studies demonstrated that the rs738409-G allele, which predisposes to NAFLD, confers modest protection from CAD (Liu et al., 2017; Simons et al., 2017). A plausible explanation for this apparent paradox may be the function of the gene product. It has been proposed that the true function of PNPLA3 is in lipid droplet remodelling and VLDL production (Trépo et al., 2016), and *PNPLA3* variants are associated with lower plasma lipid concentrations for both triacylglycerols and LDL cholesterol (Liu et al., 2017), explaining the negative correlation between the polymorphism and CAD. The rs738409-G polymorphism for *PNPLA3* (Ile148Met) reduces hydrolyse activity, impairing intrahepatic TG breakdown which hampers VLDL particle production and secretion (Anstee and Day, 2015). Therefore, the *PNPLA3* minor allele is correlated with reduced plasma lipids (Tang et al., 2015), and the negative association between *PNPLA3* and CAD.

However as indicated, the same I148M polymorphism is associated with the risk of developing NASH, as well as advanced fibrosis and cirrhosis. The possible mechanisms for this association are that the wild-type PNPLA3 protein hydrolyses TG and retinyl esters, whilst the rs738409-G variant results in a loss of function, causing TG accumulation and retinyl esters in lipid droplets in hepatic stellate cells and hepatocytes (Huang et al., 2011; Pingitore et al., 2014; Pirazzi et al., 2014). This predisposes the liver to cellular injury, and hinders extracellular protein release in the hepatic stellate cells that has beneficial effects in preventing fibrosis progression, portal hypertension and tumorigenesis (Pingitore et al., 2016). Moreover, hepatic stellate cells carrying *PNPLA3* variant demonstrated activated Yap/Hedgehog pathways, enhancing the altered anaerobic glycolysis and synthesis of Hedgehog and Yap signalling (Bruschi et al., 2020). PNPLA3 I148M isoform can also further prevent the binding of α/β hydrolase domain-containing 5 (ABHD5) and Adipose Triglyceride Lipase (ATGL) as it escapes ubiquitylation, that plays a role in TG hydrolysis (Basu Ray, 2019). With PNPLA3-I148M overexpression, the lysophosphatidic acid acyl transferase activity increases, suggestive of a gain-of-function mutation that promotes lipid synthesis (Mcmahon et al., 2014). This variant has also been positively correlated with alcoholic liver diseases, chronic hepatitis C-related cirrhosis and hepatocellular carcinoma (Bruschi et al., 2017). The significant association between *PNPLA3* rs738409-G and NAFLD was first reported by Romeo et al. (2008). The frequency of *PNPLA3* I148M is higher in the Hispanic population, and lower in European Americans and African Americans (Bruschi et al., 2017). It is also associated with hepatic fat, independently replicated in several studies of various ancestries and geographical disparate cohorts (Trépo et al., 2016). Sookoian and colleagues confirmed the strong link between I148M *PNPLA3* and NAFLD severity, determined by liver biopsy, after adjusting for important confounders such as body mass index, age, sex and insulin sensitivity. This genetic variant was not only associated with the increased risk of simple steatosis, but also NASH, advanced fibrosis and cirrhosis in the Japanese, Italians, Malaysians and Americans populations (Valenti et al., 2010a; Valenti et al., 2010b; Hotta et al., 2010; Rotman et al., 2010; Zain et al., 2012). A further interesting observation was that the recurrence of NASH post-transplantation was significantly linked to the donor genotype, rather than the host. With higher prevalence of NASH recurrences in liver recipients who received the homozygous I148M allele, this suggests that *PNPLA3* exerts its core physiological function primarily in the liver (Miyaaki et al., 2018). Reports also highlight differences in the lipid profiles between “metabolic NAFLD” and “*PNPLA3* NAFLD”, with higher levels of polyunsaturated TGs in the genetic “*PNPLA3* NAFLD” compared to the metabolic-related NAFLD, thus proposing that these two entities may be distinct drivers of fatty liver diseases.

### Transmembrane 6 superfamily 2 gene

The rs58542926-T allele of the *TM6SF2* gene has cardioprotective effects (Dongiovanni et al., 2015a; Simons et al., 2017). TM6SF2 is involved in VLDL production, and variants of the gene are associated with reduced plasma LDL-cholesterol and triacylglycerols (Liu et al., 2017). Similar to the *PNPLA3* polymorphism, the *TM6SF2* SNP has a negative relationship with CAD.

Previous GWAS reported *TM6SF2* SNPs associated with increased risk of NAFLD. TM6SF2 acts as a liver fat metabolism regulator, affecting TG secretion and hepatic fat droplet contents (Mahdessian et al., 2014). The *TM6SF2* rs5854926 coding variant (*TM6SF2* E167K) is associated with reduced *TM6SF2* expression in the liver, higher serum alanine aminotransferase levels and TG content, as well as reduced total cholesterol and LDL (Holmen et al., 2014; Kozlitina et al., 2014; Dongiovanni et al., 2015b; Grandone et al., 2016). As NAFLD is caused by hepatic accumulation of TG, its elevated levels in the liver likely mediates the increased risk for the rs58542926-T allele (E167K) (Donnelly et al., 2005). Indeed, this allele is also an independent risk factor for liver steatosis (Kozlitina et al., 2014; Sookoian et al., 2015; Grandone et al., 2016). A meta-analysis revealed that the *TM6SF2* rs5854926 was significantly associated with NAFLD in both the Asian and Caucasian populations (Chen et al., 2019).

## Cardiomyopathy

### Tumour necrosis factor

The tumour necrosis factor-α (TNF-a) encodes a multifunctional proinflammatory cytokine that is part of the TNF superfamily. Its encoding gene is located in the short arm of chromosome 5 in the major histocompatibility complex class III region. Mostly secreted by macrophages, TNF is involved in the regulation of a large range of biological processes such as cell proliferation, differentiation, apoptosis and lipid metabolism (El-Tahan et al., 2016). The role of TNF-a in the pathophysiology of congestive heart disease (Levine et al., 1990; Mcmurray et al., 1991; Dutka et al., 1993; Katz et al., 1994). Has been reported in several studies. As genetic polymorphisms in *TNF* locus is related to inflammatory disease processes, the *TNF-a* gene polymorphisms and their associations with dilated cardiomyopathy (DCM) are of emerging interest (Kubota et al., 1998; Ito et al., 2000; Tiret et al., 2000). However, to date, there are conflicting data on the association between *TNF-a* polymorphisms and DCM. Alikasifoglu et al. (2003) examined Turkish patients with DCM but was not able to demonstrate any associations between *TNF-a/-*238 and -308 (G/A) polymorphisms and DCM, in parallel with several other findings (Kubota et al., 1998; Tiret et al., 2000). There were also no associations between *TNF-a* polymorphisms and ischemic heart disease (Herrmann et al., 1998). On the contrary, a recent study reported that the *TNF-a* promoter variant 2 (TNF2) (*TNF-a*/-308 A allele) was enriched in patients with end-stage non-ischemic DCM (Densem et al., 2002). Another report on heart transplant patients with severe symptomatic myocardial dysfunction found a higher proportion of *TNF2* allele carriers in those with non-ischemic aetiologies, compared to those with ischemic cardiomyopathy. Those with non-ischemic cardiomyopathy had higher prevalence of *TNF2* compared to healthy individuals (Densem et al., 2002). These preliminary findings propose a genetic predisposition in a small subgroup of patients who may potentially respond favourably to anti-TNF-a therapy (Densem et al., 2002).

A postulated reason for this discrepancy may be the different inclusion criteria of cardiomyopathy, with several studies that reported the lack of association between *TNF-a* polymorphisms and DCM mainly included patients with New York Heart Association (NYHA) class II or III, with only a small number of patients with end-stage heart failure. Moreover, due to the high mortality associated with end-stage heart failure, a sizeable proportion of patients may not have reached the hospital and therefore opportunities were lost in screening for the *TNF-a* polymorphism (Alikasifoglu et al., 2003). Another explanation may be the variability of *TNF-a* polymorphisms associated with DCM genesis in the different ethnic cohorts. For instance, Ito and colleagues reported that the *TNF-a*/-308 A allele was more prevalent in Japanese patients with idiopathic DCM (Ito et al., 2000), which was not found in the Turkish cohort (Alikasifoglu et al., 2003). Similarly, *TNF-a*/-238 (G/A) allele frequencies in the Turkish cohort differed from those in the France and Northern Ireland cohorts (Herrmann et al., 1998). Ethnic factors may have an important role in the variability of results, and further studies are needed to clarify this hypothesis.

Increased levels of TNF-α have also been associated with increased risk of NAFLD progression to NASH (Antuna-Puente et al., 2008; Marra and Bertolani, 2009), due to its correlation with insulin resistance and inflammatory milieu. The cytokine has a broad role in inflammation, autoimmunity, tumour apoptosis and metabolic dysregulation (Pfeffer, 2003). Candidate gene studies have identified several polymorphisms in the *TNF-α* gene promotor (Qidwai and Khan, 2011). The higher prevalence of the *TNF-α*/-238 promotor polymorphism in NAFLD or NASH patients, compared to controls, are reported in the Italian (Valenti et al., 2002), Mexican and Chinese (Hu et al., 2009; Zhou et al., 2010; Trujillo-Murillo et al., 2011) cohorts. In the Japanese, two other polymorphisms have instead been identified, -*1031C* and -*863A* (Tokushige et al., 2007). Of interest, there was an inverse effect on regulation of glucose and lipid homeostasis associated with the *TNF-α*/-238 polymorphism, demonstrating increased risk of impaired insulin sensitivity, but lower LDL-cholesterol and BMI (Naik et al., 2013). A genomic meta-analysis of 8 studies also confirmed the association between -238 polymorphisms in the *TNF-α* gene and NAFLD (Wang et al., 2012). The difference in the prevalence of various polymorphisms across the unique cohorts are likely underpinned by ethnic differences, the frequency of variations or lack of statistical power across different studies (Wong et al., 2008). Furthermore, *TNF-a* mRNA overexpression was also more prominent in patients with more significant fibrosis, compared to those with mild or non-existent fibrosis, thus elucidating the important role of the TNF-a system in the pathogenesis of NASH (Crespo et al., 2001).

## Atrial fibrillation

### IL 6

The interleukin-6 (IL6) gene encodes a cytokine that plays an important role in inflammation and the maturation of B cells. The encoded protein is an endogenous pyrogen that is primarily produced in areas at acute and chronic inflammation. The functioning of this gene is implicated in a large range of inflammatory diseases (Marcus et al., 2008). In fact, the mechanism by which inflammation and stimulation of C-reactive protein secretion occurring in AF remains unclear. Nevertheless, animal studies have suggested that IL6 stimulates matrix metalloproteinase-2, mediating atrial remodelling in AF (Xu et al., 2004; Luckett and Gallucci, 2007). IL6 has reported significant correlation with increased left atrial size (Psychari et al., 2005), an important predictor for new onset AF. Moreover, polymorphisms in the promotor region of *IL6* correlated with postoperative AF (Gaudino et al., 2003; Bittar et al., 2005). Cohort studies have shown that the CC genotype of the *IL6*–*174 G/C IL6* polymorphism is associated with at least 2 fold increase in AF incidence, compared to GC and GG genotypes. Moreover, the same genotype (CC) is correlated with significant elevation of IL6 levels.

The *IL6*–174C genetic polymorphism was more prevalent in NAFLD, compared to controls. C carriers in NAFLD patients had higher homeostatic model assessment for insulin resistance (HOMA-IR) and fasting insulin compared to G carriers. Cohort studies of a Caucasian population demonstrated that the prevalence of *IL6*-174C variant was higher in NASH than NAFLD patients, and was associated with increased insulin resistance (Carulli et al., 2009). This is in line with several reports that support the association of the C allele with diabetes, insulin resistance and other manifestations of metabolic syndrome (Carulli et al., 2009). This provides a better understanding of the genetic susceptibility and pathogenesis of NASH, with the *IL6*-174C polymorphism being an independent predictor of both NAFLD and NASH, and also involved in inflammation and insulin resistance (Carulli et al., 2009).

## Interactions of genetic and environmental risk factors

**FIGURE 1:**
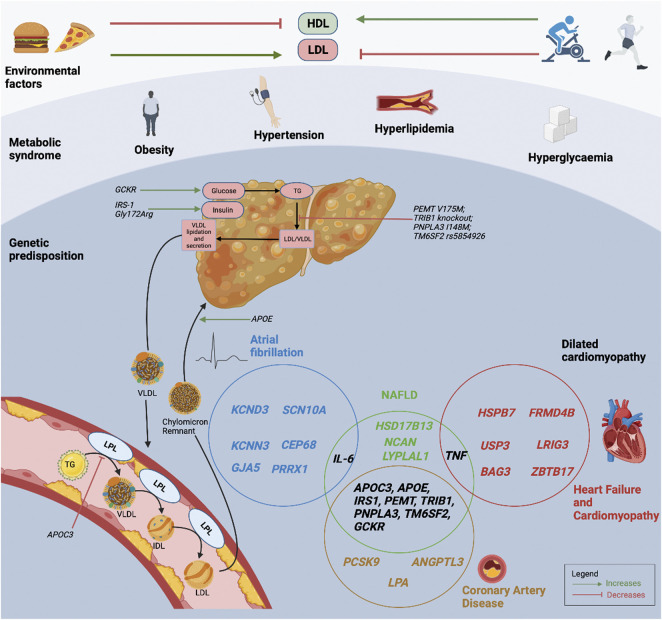
Interactions between environmental factors, metabolic profiles, and an individual’s genetic predisposition for NAFLD and CVD. The Venn diagram demonstrates the interactions between NAFLD and CVD susceptibility genes. HDL, high-density lipoprotein cholesterol; LDL, low-density lipoprotein cholesterol; LPL, lipoprotein lipase; NAFLD, non-alcoholic fatty liver disease; TG, triglyceride; VLDL, very low-density lipoprotein cholesterol.

The knowledge of genetic CVD risk may offer preventive and treatment strategies to targeted patient groups. Individuals with high genetic risks can have substantial risk reduction through pre-emptive improvement in lifestyle measures with regular moderate exercise, healthy diet and abstinence from smoking (Khera et al., 2016). The UK Biobank which examined individuals with a poor lifestyle, showed that those with higher genetic risk had more than 4-fold increase in CAD risk, compared to those with lower genetic risk. Importantly, those with high genetic risk but healthy lifestyle had lower CAD risk compared to those with low genetic risk but poor lifestyle (Said et al., 2018). Similarly, in the case of NASH, not all obese individuals with fatty liver progress to NASH, whilst conversely, some lean individuals with fatty liver do progress to NASH, emphasizing the important interactions between environmental risk factors and heritable factors (Younossi et al., 2016). However, the beneficial influence that genetic risk scores has on behavioural modifications remains lacking, as reported by a study that genetic risk information failed to influence smoking, cessation physical activity or dietary habits (Hollands et al., 2016; Jouni et al., 2017). Future prospective studies are warranted to investigate whether the knowledge of genetic risk can be translated to decrease CVD risks (Kessler and Schunkert, 2021). Figure 1 represents the interactions between environmental factors, the individual’s metabolic profile, and the genetic predisposition for NAFLD and CVD.

### Obesity and genetic risk factors

Obesity exposes the association of *PNPLA3* I148M to elevated hepatic fat levels and risk of NASH, with more pronounced impact of hepatic injury in obese individuals compared to lean individuals, and confers genetic susceptibility from a young age (Romeo et al., 2010a; Romeo et al., 2010b; Giudice et al., 2011; Palmer et al., 2012). The effect of the *PNPLA3* I148M allele on hepatic fat levels also drastically increase in patients with high visceral abdominal fat levels (Graff et al., 2013) and BMI (Stender et al., 2017). The effect of high BMI in enhancing NASH risk in *PNPLA3* I148M carriers may be mediated by insulin resistance (Barata et al., 2019). The prevalence of NASH ranged from 9% in lean 148-Ile homozygotes to 84% in obese 148-Met homozygotes (Stender et al., 2017). The obesogenic environment transforms *PNPLA3* I148M into a major determinant in NAFLD and NASH pathophysiology, predisposing these individuals to CVD events. Evidence also suggests that *PNPLA3* I148M may modify treatment response, with an effect on body weight and liver fat reduction in NAFLD patients (Carlsson et al., 2020) (i.e., lifestyle modification, bariatric surgery, omega-3 fatty acids). The interactions between obesity and *TM6SF2* E167K and *GCKR* have also been described (Azuma et al., 2009; Stender et al., 2017).

### Insulin resistance and genetic risk factors

Insulin resistance and type 2 diabetes, important risk factors for both CVD and NASH, have interactions with gene function that associate with different lipid profiles. NAFLD associated with *PNPLA3* I148M had higher levels of hepatic polyunsaturated triacylglycerols, compared to NAFLD associated with insulin resistance which had higher levels of saturated and mono-unsaturated triacylglycerols, free fatty acids and ceramides. This can lead to different lipid compositions in the liver, especially in *PNPLA3* I148M carriers, with reduced polyunsaturated triglyceride levels in VLDL particles.

*PNPLA3* I148M carriers also have an increased risk of type 2 diabetes, as reported in a large GWAS study and fine-mapping meta-analysis (Fuchsberger et al., 2016; Dongiovanni et al., 2018; Mahajan et al., 2018). In a MR study, the genetic risk score for hepatic fat accumulation revealed a casual relationship with insulin resistance, but the relationship was no longer present when the model was adjusted for liver fibrosis (Dongiovanni et al., 2018). With these findings, the authors proposed that insulin resistance is not determined by the genetically-associated high hepatic fat levels per se, but rather the insulin resistance develops as a consequence of the progression of liver disease, which may be mediated by the inflammatory and pro-fibrotic environment (Dongiovanni et al., 2018). The causes and consequences of hepatic steatosis and inflammation in NASH patients may differ between *PNPLA3* I148M carriers and those without the variant (Carlsson et al., 2020).

### Interactions of cardiovascular disease and NAFLD

The genetic cross-talk between CVD and NAFLD is therefore best exemplified with the example of the *PNPLA3* I148M polymorphism. Despite epidemiological data that advanced NAFLD is associated with increased risk of CAD, there is little evidence that hepatic fat accumulation causes atherosclerosis (Santos et al., 2019). The *PNPLA3* I148M variant correlates to only a small reduction in ischemic heart disease risk (Liu et al., 2017; Lauridsen et al., 2018) but was strongly associated with liver-related and all-cause mortality (Unalp-Arida and Ruhl, 2020). Moreover, circulating TG and LDL cholesterol concentrations can be lower or unchanged in *PNPLA3* I148M carriers compared to noncarriers (Speliotes et al., 2010; Hyysalo et al., 2014; Liu et al., 2017; Sliz et al., 2018). Nonetheless, NAFLD in itself bears increased mortality (Stender and Loomba, 2020). Therefore, the complex interplay between NAFLD and CAD is ultimately a combination of shared underlying risk factors, and putative overlapping genetic background that determines the cause and consequence of NAFLD on CVD outcomes (Carlsson et al., 2020).

## Potential targeted therapeutic approach

The progressive role of genetics in CVD and NAFLD has added knowledge to the pathophysiology of the disease entities, and offers novel therapeutic targets. It also offers the chance of identifying individuals at risk with greater precision than relying only on conventional risk scores. Polygenic risk scores have the ability of predicting those at risk in early stages before other risk factors or imaging modalities can be applied effectively. Genetics may guide prevention strategies before the development of conventional risk factors or the disease manifestation (Kessler and Schunkert, 2021). Polygenic risk scores, calculated as the summation of the number of genetic variants weighted for their effect estimate, can also be used to study the inter-relationship between the various CVD and NAFLD, and perhaps help to predict NAFLD individuals who will progress to develop CVD manifestations. Polygenic risk scores have indeed performed better for predicting CAD or MI, compared to traditional risk factors (such as smoking or hyperlipidaemia) in cohorts of various ancestry (Tada et al., 2015; Inouye et al., 2018; Khera et al., 2018; Khera et al., 2019; Wang et al., 2020).

There are several challenges with polygenic risk scores which include the transferability of results from individuals of European ancestry to other ethnicities. The concept of “missing heritability” in CVD remains an important issue, with the heritability of CAD that can be explained by currently recognised risk variants, believed to be less than approximately 30%. The feasibility of identifying variants that can explain the large portion of heritability of complex traits is still unclear. The genetic basis of complex CVD may be seen as probabilistic rather than deterministic. Moreover, the risk of CVD that is often thought to be determined by monogenic risk variants, is likely modulated by polygenic risk (Fahed et al., 2020). Guidelines on genetic risk scores in the prevention and treatment of CVD is needed to address the indication, implementation and adequate genetic counselling before these scores can be used routinely in the clinical setting.

Moreover, the potential impact of genetic variants on the treatment of NASH can be encapsulated by the example of *PNPLA3* I148M variant and the response to treatment (Sanyal et al., 2015; Wattacheril et al., 2018). At present, there are no pharmacological therapies for NASH treatment, but guidelines recommend weight reduction through lifestyle measures. However, lifestyle interventions are often limited, short-term and ineffective (Baran and Akyüz, 2014; Gitto et al., 2015). Metabolic bariatric surgery decreases hepatic steatosis, steatohepatitis and fibrosis (Laursen et al., 2019; Lee et al., 2019). Hence, it has been noted that *PNPLA3* I148M carriers had more effective reduction in hepatic fat levels with lifestyle interventions and bariatric surgery, compared to non-carriers (Shen et al., 2015; Krawczyk et al., 2016a; Krawczyk et al., 2016b). However, omega-3 fatty acid treatment was reportedly less effective in reducing hepatic fat levels in *PNPLA3* I148M carriers, compared to noncarriers in randomised trials (Nobili et al., 2013; Scorletti et al., 2015; Eriksson et al., 2018; Oscarsson et al., 2018).

Overall, genetic information is key for precision medicine. The goal of polygenic risk scores is to assist individuals with changing their lifestyle and modifiable behaviour, as well as to make informed decisions on preventive pharmacological (e.g., lipid-lowering) or surgical (e.g., bariatric) therapy in the effort for primary prevention (Kessler and Schunkert, 2021).

## Conclusion

Over the past decade, tremendous effort has been taken to elucidate the genetics of CVD and NAFLD. This has provided exciting insights to CVD and NAFLD pathophysiology, and the increasing awareness of the genetic cross-talk between the two pathologies. Indeed our understanding of the overlap remains incomplete. More novel ground-breaking treatment targets such as proprotein convertase subtilisin/kexin type 9 (PCSK9) may be forthcoming, and a breakthrough in NAFLD and/or CVD prevention and reduction will be the next important milestone. The emergence of polygenic risk scores reflects the anticipation surrounding the power of genetics and precision medicine, in improving risk prediction, personalising prevention and treatment strategies.

## Key points

• There are several non-alcoholic fatty liver disease (NAFLD) susceptibility genes that have colinear correlations with cardiovascular diseases including coronary artery disease, cardiomyopathy and atrial fibrillation
• Certain NAFLD susceptibility genes, such as phospholipase domain-containing protein 3 and transmembrane 6 superfamily 2 that regulate VLDL particle production, have cardioprotective effects for coronary artery disease.
• Genetic and environmental risk factors have complex interactions that lead to disparity in genetic influence on NAFLD and incident cardiovascular disease.
